# Observational study of patients with occipital condyle fracture at a brazilian referral trauma center

**DOI:** 10.1590/0100-6991e-20213024

**Published:** 2021-11-19

**Authors:** ANDREW VINÍCIUS DE SOUZA BATISTA, GUILHERME BRASILEIRO AGUIAR, PRISCILLA BENNETT, MÁRCIA RAMOS UMIGI, JOSÉ CARLOS ESTEVES VEIGA

**Affiliations:** 1 - Santa Casa de São Paulo School of Medical Science, Neurosurgery - São Paulo - SP - Brasil

**Keywords:** Craniocerebral Trauma, Basilar Skull fracture, Traumatic Brain injuries, Atlanto-Occipital Joint, Traumatismos Craniocerebrais, Fratura da Base do Crânio, Lesões Encefálicas Traumáticas, Articulação Atlantoccipital

## Abstract

**Objective:**

to evaluate the clinical-epidemiological characteristics, treatment, and evolution of patients with occipital condyle fracture (OCF) at one of the largest referral trauma centers in Latin America.

**Methods:**

this was a retrospective observational study of OCF identified from trauma cases admitted between December 2011 and December 2019 by the neurosurgery team at a Type 3 trauma center.

**Results:**

a total of twenty-eight occipital condyle fractures were identified in twenty-six patients. The incidence was less than 0.2% per year and more common in male patients (4:1 ratio) involved in traffic accidents. The mean age was 42.08 years. Anderson and Montesano type II and Tuli type 1 were the most frequent (67.9% and 89.3%, respectively) and no case presented C0-C1-C2 instability. All patients were treated with a cervical collar for 3 to 6 months. About 65% of the patients exhibited good progression (Glasgow Outcome Scale equal to 4), and the severity of traumatic brain injury was the main determinant for negative outcomes.

**Conclusion:**

the findings of this study are in accordance with available literature data. The use of external stabilization with a cervical collar is reinforced for the treatment of stable lesions, even when these are bilateral. Assessment of the patients’ follow-up results in the studied sample may contribute with useful information for the treatment of occipital condyle fractures.

## RESUMO

**Objetivo:**

avaliar o perfil clínico-epidemiológico, o tratamento e a evolução de pacientes com fraturas do côndilo occipital (FCO) em um dos maiores centros especializados em trauma na América Latina.

**Método:**

este é um estudo observacional retrospectivo de FCO identificadas em casos de trauma que foram atendidos no período de Dezembro de 2011 a Dezembro de 2019 pela equipe de trauma de centro de trauma Tipo 3.

**Resultados:**

um total de vinte e oito fraturas do côndilo occipital foram identificadas em 26 pacientes. A incidência foi inferior a 0.2% ao ano e mais comum em pacientes do sexo masculino (proporção 4:1) envolvidos em acidentes de trânsito. A idade média foi de 42.08 anos. O Tipo II de Anderson e Montesano e o Tipo 1 de Tuli foram os mais frequentes (67.9% e 89.3%, respectivamente) e nenhum caso teve instabilidades C0C1C2. Todos os pacientes foram tratados com colar cervical por período de 3 a 6 meses. Cerca de 65% dos pacientes apresentaram boa evolução (Escala de Resultados de Glasgow maior ou igual a 4), e a gravidade da lesão cerebral foi o principal determinante para os resultados negativos.

**Conclusão:**

os achados deste estudo são similares a dados disponíveis na literatura. O uso de colar cervical para estabilização externa é reforçado para o tratamento de lesões estáveis, mesmo quando bilaterais. A avaliação dos resultados do acompanhamento dos pacientes na amostra estudada pode contribuir com informações úteis para o tratamento de fraturas de côndilo occipital.

## INTRODUCTION

Occipital condyle fractures (OCF) were initially described in the context of cadaveric anatomical studies, with the first record dating back to the first half of the 19^th^ century^1^. The number of reported cases in the literature before computed tomography became available is less than ten^2^, and this can be related to the difficulty in identifying such alterations in simple radiographic images. Only with the development of more accurate imaging methods it was possible to accurately diagnose and study fractures involving the occipital condyle. Still, this entity is considered rare, even at specialized centers^3^ ^-^ ^5^. The diagnosis thereof should be suspected when blunt trauma secondary to a high-energy mechanism, altered consciousness, cervical pain, restricted movement of the neck, lower cranial nerve dysfunction, and edema of retropharyngeal soft tissues are either reported or present^6^.

**Figure 1:**
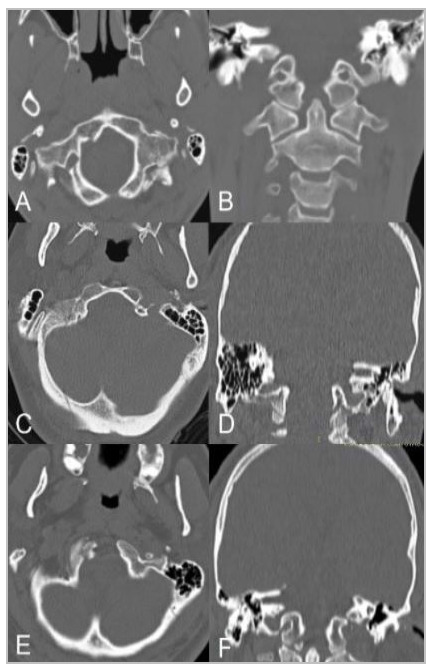
Examples of occipital condyle fractures. The first line shows the axial (A) and coronal (B) sections of a patient with A&M Type I OCF on the right side (Tuli Type I). The second line (C and D), in turn, shows a linear occipital fracture on the right side extending to the ipsilateral occipital condyle (A&M Type II, Tuli Type I). The last line (E and F) shows an example of avulsion of an occipital condyle fragment (A&M Type III, Tuli Type IIA). Fractures are demonstrated by arrows. OCF: fracture of the occipital condyle; A&M: Anderson and Montesano. Source: Authors.

In 1988, Anderson et al.^2^, classified OCF according to their morphology and injury mechanism, based on a retrospective analysis of six patients, and it is still the most used classification worldwide when characterizing OCFs (Figure 1). Type I fractures are comminuted, with minimal or no misalignment of bone fragments, and they are the result of an axial force imposed to the condyle. Type II includes cases in which the OCF is an extension of a skull base fracture, secondary to direct and local impact. Type III corresponds to the avulsion of a fragment of the inferior and medial aspects of the condyle towards the dens of the axis; in this case, the biomechanics of the trauma usually consist of lateral rotation and/or flexion of the cervical region, both of which promote an increased tension on the alar ligament^2^.

More recently, Tuli et al.^7^ proposed a new classification system based on the degree of ligament injury due to the fracture, suggested by fragment displacement and identification of occipital-cervical instability in imaging findings and based on criteria defined by the authors (Figure 1). Thus, Type 1 OCF are those in which the fragments are not displaced and are considered stable (which includes Anderson et al.^2^ Types I and II); Type 2 OCF are characterized by the dislocation of bone elements and are subdivided into 2A, when there is no occipital-cervical instability, and 2B, when the O-C1-C2 segment is unstable^7^. Like Tuli et al.^7^, other authors have tried to establish criteria for studying the stability of the craniocervical junction and, thus, provide guidelines for the treatment of these cases.

Although there is no consensus on the best treatment, most patients are treated conservatively with a cervical collar, which leads to satisfactory outcome, in clinical practice. This study aimed to describe a sample of occipital condyle fractures at a Brazilian specialized trauma center and compare them with available literature data.

## METHOD

This is a retrospective observational study conducted between December 2011 and December 2019. The convenience sampling method was used with trauma cases treated at the emergency unit of Central Hospital of Santa Casa de Misericórdia de São Paulo, a Type 3 trauma center. All patients who met the criteria for undergoing a computed tomography scan of the skull and/or cervical spine were assessed for the presence or absence of OCF. Twenty-seven cases were identified, but one was excluded due to insufficient information in the medical record.

The data referring to trauma epidemiology, the patients’ clinical and neurological status, and the procedures adopted by each assisting team were obtained from medical records retrospectively. The information related to the characterization of injuries resulting from the trauma, in turn, were collected by the analysis (by two different medical professionals) of images (radiography, tomography, and magnetic resonance images) and the official reports issued by radiologists. Condyle fracture consolidation was not found in most patients, which made the analysis of this variable unfeasible.

Statistical analysis of the data was performed using the Statistical Package for Social Sciences (SPSS) software version 21 for Windows®. The study was approved by the Research Ethics Committee of the institution (CAAE 20413513.9.0000.5479; approval document number 470.097).

## RESULTS

### Sample characterization

**Figure 2:**
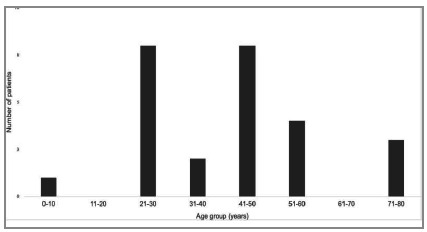
Distribution of patients by age group.

Data from twenty-six patients having OCF were analyzed (Table 1). Twenty-one were males, and the mean age was 42.08 ± 18.42 years (Figure 2). It was not possible to determine the exact prevalence of OCF in the studied population, but it is estimated that it is less than 0.2% and that the incidence is less than 2/1.000 trauma patients per year as based on the institution’s records of trauma cases^8^.

**Table 1:** Demographics, characterization of traumatic injuries, and patients’ follow-up.

Variables	n (%)
Sex*	
Male	21 (80.8)
Female	5 (19.2)
Trauma mechanism	
Fall from height	11 (42.3)
Hit by an automobile	8 (30.8)
Motorcycle accident	7 (26.9)
TBI severity	
Mild (GCS 14-15)	14 (53.8)
Moderate (GCS 9-13)	4 (15.4)
Severe (GCS 3-8)	8 (30.8)
OCF Type (Anderson et al.)	
Type I	6 (21.4)
Type II	19 (67.9)*
Type III	3 (10.7)
OCF Type (Tuli et al.)	
Type 1	25 (89.3)*
Type 2	
2A	3 (10.7)
2B	0
OCF Laterality	
Right side	14 (53.8)
Left side	10 (38.5)
Bilateral	2 (7.7)
Presence of other intracranial injuries	20 (76.9)
Presence of extracranial injuries	16 (61.5)
Associated cervical spine fracture	2 (7.7)
GOS at the time of hospital discharge	
1 (death)	8 (30.8)
Cause of death	
TBI	6 (75)
Infection	2 (25)
2	0
3	1 (3.8)
4	2 (7.7)
5	15 (57.7)

*Condyles were counted separately in cases with bilateral fracture

TBI: traumatic brain injury; GCS: Glasgow Coma Scale; OCF: occipital condyle fracture; GOS: Glasgow Outcome Scale.

### Characterization of trauma

The most commonly associated trauma mechanisms were falls from height, followed by hit by car and motorcycle accident. It was not possible to verify association between the trauma mechanism and the type of condyle fracture.

Head trauma was considered mild in 53.8% of the cases and severe in 30.8%. In addition to the condyle fracture, other injuries were found on cranial computed tomography images in 76.9% of the patients, three of whom required urgent neurosurgical treatment due to brain injury.

As for extracranial injuries, these were found in 16 patients (61.5%), of whom nine were referred to interventional treatment by the general or orthopedics surgeons. Three patients had associated spinal fractures, with the cervical segment being the most affected (two patients had compression fractures of C2).

### Neurological clinical presentation

The mean value in the Glasgow coma scale when admitted to the emergency room was 10.6. Two patients had anisocoria, one of whom having suffered cardiorespiratory arrest during prehospital care.

The most frequently observed neurological signs and symptoms were changes in the level of consciousness, headache, and cervical pain. No abnormalities were described for any patient when examining the cranial nerves.

### Condyle fracture characterization

The evaluation of the computed tomography scans of the twenty-six patients led to the identification of twenty-eight OCF, given that in two cases both condyles had been affected. In most cases, the affected side was the right side (53.8%, excluding cases of bilateral fracture).

Anderson et al.^2^, Type II and Tuli et al.^7^, Type I were the most prevalent from each classification system, accounting for 67.9% and 89.3% of fractures, respectively. In one case, the fracture involved the hypoglossal nerve canal. None of the fractures were considered unstable (Tuli et al.^7^, 2B), as there were no cases of medullary compression clinically expressed or documented by imaging techniques.

Type II fractures showed a statistically significant association between severe traumatic brain injury and death, but not with the existence of other extracranial injuries secondary to the trauma (Table 2). It was not possible to establish a statistically significant relationship between OCF type and trauma mechanism.

**Table 2:** Association between traumatic brain injury severity and occipital condyle fracture type.

Variables	OCF Classification (Anderson et al.)			Total	p-value*
	Type I	Type II	Type III		
TBI Classification					
Mild	6	5	3	14	0.017
Moderate	0	4	0	4	
Severe	0	8	0	8	
Presence of ECI					
Yes	4	10	2	16	0.999
No	2	7	1	10	
Clinical outcome					
GOS 1 (death)	0	8	0	8	0.045
GOS >1	6	9	3	18	

*After applying Fisher’s exact test, with a 95% confidence interval. OCF: occipital condyle fracture; TBI: traumatic brain injury; ECI: extracranial injury; GOS: Glasgow Outcome Scale.

### Condyle fracture treatment

Considering that no case was classified as unstable, all patients underwent non-surgical treatment. External immobilization with Philadelphia-type cervical collar was worn for a period ranging from 3 to 6 months.

### Follow-up and outcome

Follow up of the patients was hampered due to an excessive number of dropouts during follow-up, therefore, it was impossible to adequately assess this variable.

The in-hospital mortality rate was 30.8%, with traumatic brain injury being the cause of 75% of deaths. In average, these patients had a score of 4.6 in the Glasgow coma scale upon admission to the emergency unit. Patients who died had type II fractures, and these were associated with the presence of intracranial hemorrhagic collections seen in cranial computed tomography scans. In addition, 75% of them had traumatic extracranial injuries and, in 62.5% had been hit by a car.

Patients who showed good neurological results (having a Glasgow Outcome Scale score greater than or equal to 4) made up 65.4% of the sample. One case had severe sequelae at hospital discharge. All fractures classified as Anderson et al.^2^, types I and III, in turn, had a score in the Glasgow Outcome Scale equal to 5 at the time patients were discharged from the hospital.

As for OCF-related complaints, almost all patients who reported having cervical pain upon hospital admission showed improvement once underg analgesia and after wearing a cervical collar. Only one patient needed prolonged analgesia for persistent cervical pain, despite the absence of radiological instability during follow-up as an outpatient. No new neurological deficits were seen for these patients.

## DISCUSSION

The increasing availability and use of diagnostic imaging techniques have, indeed, led to an increase in the identification of OCF, to the point that Ciappetta et al.^9^ currently consider it a common injury. However, most authors recognize this type of injury as an infrequent one. Hanson et al.^4^, indicate a prevalence of 0.1-0.2% among patients with severe polytrauma, whereas Mueller et al.^5^ estimate that at least 1% of patients in a high complexity trauma center are expected to have OCF. A Brazilian series identified this injury in 16.22% of trauma cases involving the craniocervical junction that were evaluated between 2010 and 2013^10^. In contrast, in another series with 438 postmortem CT scans from trauma victims that were retrospectively analyzed the prevalence of OCF was 22.6%^11^. Our data are compatible with those published by Malham et al.^12^, who reported an incidence of 1.7 OCF cases per 1,000 patients per year.

The demographic characteristics of patients with OCF in our study are similar to those reported by others. In the literature, there is a male predominance (2:1-5:1)^2^ ^,^ ^4^ ^,^ ^11^. However, when evaluated separately, the OCF rate among trauma victims is not statistically different from that observed in females^11^. The most affected patients have a mean age between 20 and 50 years^2^ ^,^ ^4^ ^,^ ^7^ ^,^ ^13^. As for the pediatric population, the average age reported in a series of fifteen cases was 10 years^14^.

The most often listed causes of trauma underlying OCF cases involve traffic accidents^4^ ^,^ ^7^ ^,^ ^11^ ^,^ ^15^, which is in agreement with our findings if we group the cases of victims who were hit by a car or were involved in motorcycle accidents. Bearing in mind that these mechanisms are normally associated with significant systemic injuries, including intracranial ones, the presence of OCF is considered a marker of high energy trauma^3^ ^,^ ^4^ ^,^ ^13^. This observation explains the findings of multiple injuries in most patients. In our study, it was not possible to determine the severity of multiple traumas using consolidated scores such as ISS (Injury Severity Score). In the series reported by West et al.^13^, the average value of this index was 23.2.

It should be noted, however, that OCF can occur even as a result of low-energy trauma such as falls from own height. In addition, not all patients have associated severe intracranial injuries. In more than half of our sample, for example, traumatic brain injury was considered mild (based on the Glasgow coma scale) upon admission to the emergency room. These data should draw attention to the need of suspecting a diagnosis of OCF even in individuals who are clinically well. Given the accuracy of imaging devices in detecting small lesions, there are currently fewer patients with neurological deficits as compared to previous reports and case series^12^ ^,^ ^13^ ^,^ ^15^. Our findings are in accordance with those described by West et al.^13^ and Maserati et al.^15^, with no deficits due to compression of neural structures attributable to OFC.

In contrast, a considerable number of previous studies describe different clinical presentations^7^ ^,^ ^16^ ^-^ ^18^. Some oligosymptomatic forms may exhibit mild to moderate cervical pain^17^ ^,^ ^18^. Yet, there are reports of catastrophic progression due to secondary injuries associated with the fracture, such as posterior fossa hematomas, which can produce brainstem compression syndromes or upstream obstructive hydrocephalus^19^. In addition, the involvement of other cervical structures is also described, as in cases with retropharyngeal hematomas^20^. The presence of spinal cord contusion appears to be more common in children^14^.

The involvement of one or more lower cranial nerves can occur, unilaterally or bilaterally^7^ ^,^ ^21^ ^,^ ^22^ (as in Collet-Sicard syndrome)^23^, especially during the acute phase, with the hypoglossal nerve being the most commonly affected nerve (up to 74% of cases)^24^. The late appearance of cranial nerve deficit may be indicative of fractured bone fragment displacement^24^.

The diagnosis of OFC should be made preferably by means of computed tomography of the craniocervical junction (level II, American Association of Neurological Surgeons - AANS)^3^. The imaging technique allows the physicians to evaluate fracture morphology and helps them predict the existence of local instability. Magnetic resonance imaging is useful for checking the integrity of ligaments (level III, AANS)^3^ and should be requested mainly in cases with significant risk of displacements among bone fragments. In that situation, the study of atlantoaxial biomechanics by means of dynamic radiographs is also of value.

The most common fractures found among our series were Anderson et al.^2^, type II, as observed by Maserati et al.^15^ and Borowska-Solonynko et al.^11^ (41% and 75.7% of cases, respectively). Hanson et al.^4^, classified approximately 75% of their sample as condyle avulsions (type III), a value not reproduced among the largest series in our investigation. As stated by these authors, it is sometimes difficult to differentiate between non-comminuted type I OCF and type III fractures with a small displacement of the fragments^4^, which gives rise to differences among examiners.

Although the Anderson et al.^2^ system is the most often commonly used, its classification surrounding fracture morphology is not always sufficient to precisely determine stability at the craniocervical junction level, which may be detrimental to define the best therapeutic approach. When proposing a new classification model, Tuli et al.^7^ criticize, for example, the practical inapplicability of the distinction between types I and II by Anderson et al.^2^, whose treatments are the same^7^.

In an attempt to systematize the management of OCF, other authors have suggested new classification systems, also centered on the stability at the craniocervical junction. Mueller et al.^5^ categorized the OCF into three types based on the involvement of unilateral or bilateral fractures and also based on the presence of atlanto-occipital instability. Hanson et al.^4^ suggest that the presence of bilateral fracture can be used as a marker of instability, in addition to other available data such as atlanto-occipital dissociation >2mm or atlantoaxial dissociation >3mm.

According to the Tuli et al.^7^, algorithm type I fractures (without displacement) do not require supplementary testing other than tomography or stabilization. Fractures with displacement should be further analyzed regarding biomechanics. In the case of stable fractures (2A), there is clear indication to apply a rigid collar; whereas in the event of unstable fractures (2B), a halo vest or operation are indicated instead^7^. In our study, patients were assessed for the presence of instability, which was not seen in any of them. This is why they underwent conservative treatment with a Philadelphia-type cervical collar. West et al.^13^ treated 82.6% of their cases with a rigid cervical collar, while the other patients were kept under surveillance.

In a review of the AANS guidelines, forty-three of 259 listed patients did not initially receive treatment for OCF. From those, nine (20.9%) of them developed deficits within days to weeks (four had type II fractures and the other four had type III fractures). Out of the 190 cases treated with a cervical collar, sixty-eight (of whom there was follow-up information) showed complete recovery. Therefore, among the guideline recommendations, the use of external cervical immobilization for all types of OCF (level III) is postulated^3^.

A systematic English review compared the results from 25 studies encompassing 240 patients treated with external immobilization. Proportionally, the halo vest was the most used method for an average time of 11.7 weeks. Eighty patients wore the semi-rigid collar and 63, the rigid collar. Most patients had a good progression or exhibited a mild to moderate deficit, with no statistical difference between the two types of immobilizations regarding their neurological clinical outcomes^25^. Additionally, Mueller et al.^5^ did not find any significant differences in any of the three types of Anderson et al.^2^, as the fractures treated with a rigid cervical collar had the same outcomes in terms of ISS, mortality rate and radiological and clinical progression (SF-36).

Indications for surgical treatment may vary according to experts’ opinions. Vaccaro et al.^26^ even defended the primary indication of occipitocervical fixation for Type III OCF. Currently, the surgical treatment is reserved only for those occasions when there is evidence of atlanto-occipital ligament lesion, instability of the craniocervical junction (level III, AANS)^3^, or compression of neurovascular structures, in which the surgical approach must be performed urgently. It is also important to highlight the existence of concomitant fractures of the cervical spine, which can contribute to the instability of the segment. However, the occurrence of cervical fractures with biomechanical importance does not seem to be frequent^13^.

In the last review published by the AANS, seventeen surgically treated cases were mentioned, and the occipitocervical fixation was the procedure of choice in more than 82.3% of patients. As to the other remaining cases, the operation was indicated for decompression of the brainstem in fractures of types II and III^3^. On the other hand, regarding the bilateral OCF, there are controversies concerning the therapy of choice. As mentioned, the potentially unstable nature of these lesions is reinforced, and the integrity of the alar ligament and the tectorial membrane should be investigated on both sides^3^ ^,^ ^4^. Some authors have already reported satisfactory outcomes following non-surgical treatment^15^ ^,^ ^27^. Nevertheless, more rigid cervical immobilization is recommended (level III, AANS)^3^.

Overall, the prognosis of OCF per se is favorable, with good functional recovery. An observational study of 31 cases, followed prospectively for one year, showed that the quality of life was not affected by OCF but rather by comorbidities and the severity of trauma^5^. In our series, the unfavorable progression of some patients seems to be related to the severity of the traumatic brain injury. As attested, type II was statistically associated with this outcome, possibly due to the direct incidence of the trauma energy onto the skull base.

Occipital condyle fractures are uncommon injuries to the craniocervical junction and usually related to high-energy trauma. The related clinical condition commonly varies among patients, so OCF should be suspected even in oligosymptomatic patients. The diagnosis is confirmed by a computed tomography exam, which allows the analysis of fracture morphology and provides guidance to the appropriate treatment. This is further enhanced with the aid of supplementary imaging methods such as magnetic resonance. A significant portion of patients evolves well with cervical immobilization wearing a rigid collar, but in cases with atlanto-occipital instability, a halo vest and operation for internal fixation should be assessed. Considering that the available information in the literature regarding occipital condyle fracture is based mainly on experts’ opinions and, therefore, there is no solid evidence regarding the best treatment, further studies are needed.
